# The G to A transformation of rs4702 polymorphism in 3’UTR of FURIN reduced the risk of radiotherapy‐induced cognitive impairment in glioma patients

**DOI:** 10.1111/jcmm.17074

**Published:** 2021-12-24

**Authors:** Sen Yang, Zhan‐Zhao Fu, Yan‐qiu Zhang, Bao‐hong Fu, Lixin Dong

**Affiliations:** ^1^ Department of Radiotherapy (Ward II) Qinhuangdao First Hospital Qinhuangdao China

**Keywords:** FURIN, glioma, intestinal flora, radiotherapy‐induced cognitive impairment, rs4702, SNP

## Abstract

The G allele of rs4702 polymorphism has been reported to reduce the production of mature BDNF and FURIN, both of which were closely associated with cognitive functions. Real‐time PCR, ELISA and luciferase assay were performed to explore the interactions between miR‐338‐3p, FURIN and BDNF. T‐RFLP was used to assess the intestinal flora in the stool samples of glioma patients after radiotherapy. We grouped the 106 glioma patients recruited according to the rs4702 polymorphism. The results showed no obvious correlation between rs4702 polymorphism and the expression of miR‐338‐3p. However, rs4702‐A was associated with increased expression of FURIN and BDNF in the serum and PBMC of glioma patients after radiotherapy. Besides, the study found that rs4702‐A was remarkably associated with increased enterotype I and decreased enterotype III in the stool of glioma patients after radiotherapy. Rs4702‐A was also proved to be closely associated with increased MMSE, role functioning and social functioning at three months after radiotherapy. Furthermore, miR‐338‐3p repressed the expression of FURIN‐G. Compared with G allele, the presence of A allele of rs4702 polymorphism in FURIN could obstruct the suppressive effect of miR‐338‐3p upon the expression of FURIN and BDNF in intestinal flora. Therefore, the carriers of A allele will be challenged with less risk of radiotherapy‐induced cognitive impairment.

## 1 INTRODUCTION

Radiotherapy (RT) has been very effective therapeutic strategy for nasopharyngeal carcinoma (NPC) by improving overall survival rate. However, during the radiotherapy different parts of the brains are exposed to high dose radiation which can lead to side effects. These side effects result in long‐term impairment of cognitive and nervous system.^1^

Recent studies have shown that microorganism in gut of people are important for regulation of various function of central nervous system. The microorganism in the gut include variety of species of anaerobic bacteria, fungi, and other microbes.

A complex connection between the gut microbiome and brain exist, which is responsible for variety of functions. The network consists of signalling pathways related to neuroendocrine, nervous and neuroimmune system. A clear mechanism underlying the network between the microbiome and brain is not known, but few hypothesis have been proposed. The hypothesis includes, possible transport of metabolites, toxins and proinflammatory factors produced by gut microbes, the microbial metabolites activate the endocrine cells present in nervous system.

As binding sequences of the small noncoding RNAs, known as microRNAs (miRNAs), are relatively small, these miRNAs can seldom interact with more than one downstream mRNAs by binding to the 3’‐UTR sites of the mRNA. Hence, miRNAs function as regulators of gene expressions. Different types of miRNAs present in brain can modulate physiological processes, like neurological development.^2^, ^3^

FURIN are a type of protease, which belongs to a family of mammalian subtilisin that activate several precursor biomolecules. FURIN has been known to activate growth factors, glycoproteins present on the viral envelope and adhesion receptors. Recently, it was proven that FURIN can activate brain‐derived neurotrophic factor (BDNF) from it precursor in the trans‐Golgi network.

During brain development, BDNF carries out several important functions that translate into synaptic and cognitive behaviour. BDNF is a type of protein secreted into the hippocampus region of brain. Dysregulation of expression of BDNF results in variety of pathophysiological disorders including Parkinson's disease and Alzheimer's disease.

Previous research studies have shown that BDNF binds to TrkB receptors. Administration of BDNF in lateral ventricle of the rats resulted in increased proliferation and expression of TrkB receptors. The study also showed that WBI induced decreased expression of BDNF, whereas TSA restored the normal BDNF levels. TrkB receptors are hypothesized to be involved in normal brain functions, such as memory, learning and plasticity. Cui et al investigated the effect of simvastatin and HUCBC on rats, which suffered stroke.^4^ They found that the combination therapy resulted in higher expression of BDNF‐TrkB system leading to improved brain function and better recovery from stroke in the rats.

G allele was identified by previous studies as a risk factor in genome‐wide SNP rs4702 (A/G). Therefore, the authors chose to design miR‐338‐3p, which can bind to the 3’‐UTR site of FURIN. This was proposed to reduce FURIN expression and thereby resulting in decreased levels of its effector BDNF. The authors proposed that such modification can cause differential binding of miRNA and could be the source of altered gene expression in several disease states.^5^

The study upon the relationship between intestinal flora and cognitive impairment found that the prevalence of Bacteroides was increased in patients with mild cognitive impairment.^6^, ^7^ In addition, miR‐338 has been identified as an innovative modulator in the pathogenesis of Alzheimer's disease.^8^ Moreover, it has been suggested that the G allele of rs4702 polymorphism promoted the suppressive effect of miR‐338‐3p upon the expression of FURIN, thus leading to the reduced production of mature BDNF, while both FURIN and BDNF were closely associated with cognitive functions.^5^, ^9^, ^10^, ^11^, ^12^, ^13^ In this study, by investigating the key indicators of radiotherapy‐induced cognitive impairment in glioma patients, we tried to unveil the role of G>A transformation of rs4702 polymorphism in 3’UTR of FURIN and the according signalling pathways.

## 2 MATERIALS AND METHODS

### 2.1 Human subjects sample collection

For this study, glioma patients (*N* = 279) who received radiotherapy and suffered impairment of cognitive functions were recruited. The patients were classified based on the genotype of rs7853346 and rs1799864 in PTENP1 and CCR1 respectively. The patients were grouped as CC/GA&AA group (*N* = 62), the patients that had genotype of the rs7853346 CC/rs199864 GA&AA, CG&GG/GA&AA group (N=52), the patients that had genotype of the rs7853346 CG&GG/rs199864 GA&AA, CC/GG group (N=93), the patients had the genotype of rs7853346 CC/rs199864 and the CC&GG/GG group, and the patients had the genotype of the rs7853346 CG&GG/rs199864 GG. The general characteristics of the patients, such as health status, age, bladder control, itchy skin, leg weakness, history of depression, gender, were collected and compared at three time points, first before any radiotherapy, second after 1 month following radiotherapy and third after three months following radiotherapy. To enrol the patients in the study, the authors followed a few eligibility criteria, such as age (≥ 18 years), and confirmed glioblastoma diagnosis using histological tests. The patients in the study received radiation therapy (dose 60 Gy) in combination with the everyday administration of 75 mg/m2 of TMZ, followed by 150–200 mg/m2 administration of TMZ every day for five more days.^14^ To follow‐up the cognitive functions of the patients, they were scanned using MRI about 0, 1 and 3 months after radiotherapy. Peripheral blood mononuclear cells (PBMCs) were isolated from the blood samples collected from the patient groups for subsequent analysis. Institutional ethical committee has approved the protocol of this study.

### 2.2 Classification based on enterotype and their comparisons

The patients enrolled in this study were classified based on the types of the gut microbes and the comparison table of enterotype I over non‐enterotype I, whereas enterotype III over non‐enterotype III. Compared to the patients without enterotype, the patients with the enterotype I showed higher likelihood of cerebral SVD components. Among the patients with enterotype I and non‐enterotype I, not any significant differences were found. However, the patients with non‐enterotype III, which had higher levels of gut microbiome, had a less likelihood of cerebral SVD components. In addition, there was a significant difference was observed between the patients with enterotype III and non‐enterotype III related to the hypertension and alcohol use. Overall, no significant differences were observed for the ratio of type of bacteria Firmicutes and Bacteroidetes, which is named F/B ratio. Along with these results, the male and female patients showed not a lot of difference in their enterotype.

### 2.3 Gut microbiome

A simple procedure to collect faecal samples was performed by patients or their family members. The sample was collected immediately after evacuation using a scoop and placed into the collection tube, which was store at −81°C. The samples were stored at the NCGG Biobank. After all samples were collected, the gut microbiome was analysed by terminal restriction fragment length polymorphism (T‐RFLP) analysis, using T‐RFLP analysis40. T‐RFLP analysis is a well‐established method, which uses 16S ribosomal RNA‐based tool. Specially, enterotype I included Bacteroides at >30%, enterotype II included Prevotella at >15%, and enterotype III included the remaining bacteria.

### 2.4 RNA isolation and real‐time PCR

To identify the expression of miR‐338‐3p, FURIN mRNA and BDNF mRNA in each sample, RT‐qPCR was performed. Specifically, Trizol reagent (Thermo Fisher Scientific) was applied to the complete RNA extracted from peripheral blood samples from patients from both groups in compliance with the experimental standards obtained from the handbook of the manufacturer. Then, the extracted total RNA was assayed with NanoDrop™ 8,000 Microvolume UV‐Vis Spectrophotometer (Thermo Fisher Scientific) using the instruction from the manual of the instrument obtained from the manufacturer of the machine, to establish RNA top standard. This was followed by qPCR to conduct the investigation of the quantitative changes in the target genes. The expression of miR‐338‐3p (Forward: 5′‐ATATCCTGGTGCTGAGTG′; Reverse: 5′‐ GAACATGTCTGCGTATCTC‐3′), FURIN mRNA (Forward: 5′‐GCCACATGACTACTCCGCAGAT‐3′; Reverse: 5′‐TACGAGGGTGAACTTGGTCAGC‐3′) and BDNF mRNA (Forward: 5′‐ CATCCGAGGACAAGGTGGCTTG‐3′; Reverse: 5′‐GCCGAACTTTCTGGTCCTCATC‐3′) in each sample was determined using the 2^− ΔΔCt^ method.

### 2.5 Luciferase assay

To explore the regulatory network of FURIN and miR‐338‐3p, luciferase assay was performed. The six different luciferase analysis were performed. (1) Sequence analysis by TargetScan (http://www.targetscan.org/) predicted a binding site for FURIN on miR‐338‐3p; thus, wild‐type (WT) and mutant FURIN luciferase containing vectors were formed and transfected into U251 cells with miR‐339‐3p. A brief description of luciferase assay protocol was as follows. The growth medium was removed from the plates, and the cells were rinsed with the PBS solution. Precaution was taken to not dislodge the cells. The PBS solution was removed gently, and about 20 ul/well of lysis reagent was added. To the plate containing cell lysate, 100 ul of luciferase assay reagent was added to each well. The light produced was measured, and data were collected.

### 2.6 ELISA

The abundance of FURIN and BDNF in collected stool samples was measured by using commercial ELISA assay kit (Thermo Fisher Scientific) following the recommended assay procedure provided on the manual of the assay kits. In brief, the prepared coating solution was used to coat the plates with about 100 ul of it. The plates were covered and incubated overnight at 2–8°C. The wells were aspirated and washed with >200 ul of wash buffer per well. The plates were blocked with 200 uL of blocking buffer for 1 h. After similar steps, about 100 ul of streptavidin‐HRP solution was added to each well and incubated for 30 min. The absorbance of samples at 450 nM wavelengths was measured on a Multiskan GO microplate reader in following the recommended operating protocol provided by the instrument manufacturer.

### 2.7 Statistical analysis

The results of the study were expressed as group mean with standard error of the mean. The size of the samples used for this study was based on calculations from the previously reported publications. The authors used these studies and other pilot studies to determine the sample size of the study. A two‐way repeated ANOVA was used to determine the effects of the Dex treatment on the ischaemia/reperfusion models in both animal studies and cell studies. To compare the studies of larger size, the differences were measured using the one‐way ANOVA. As the repeated measure ANOVA was used in this study, which is prone to violation of sphericity, Greenhouse‐Geisser correction was applied. Data from cognitive function and motor function procedures were determined by two‐way ANOVA, which was used to differentiate between the effects of treatment and time. For all the different assays performed in the study, including TUNEL, Western blot, real‐time PCR and brain functions, *p*‐values of *p* < 0.05 were considered significant.

## 3 RESULTS

### 3.1 rs4702‐A was associated with increased expression of FURIN and BDNF in the serum of glioma patients after radiotherapy

**FIGURE 1:**
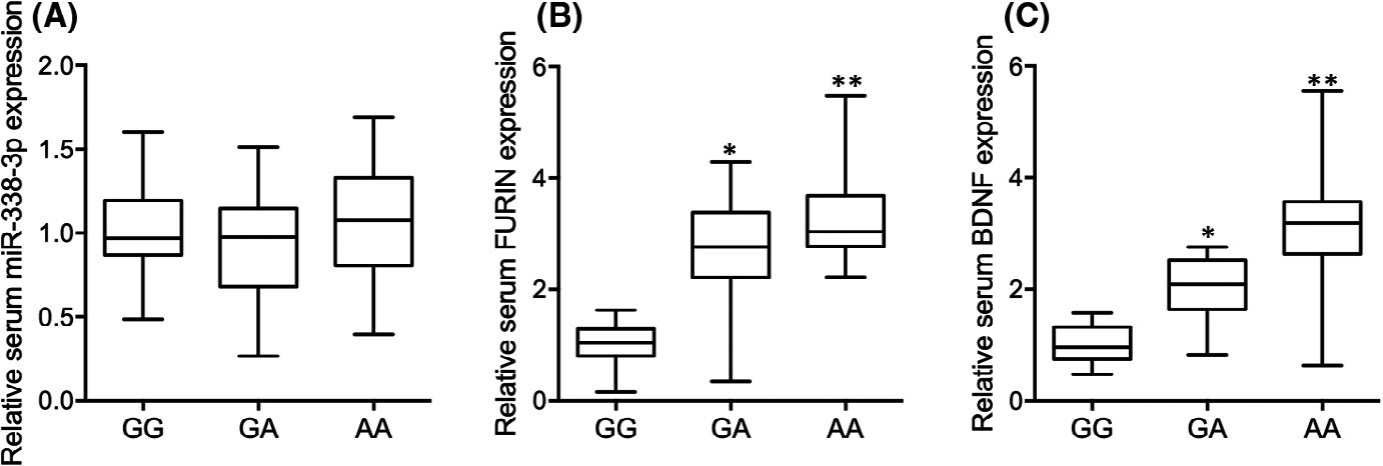
Rs4702‐A was associated with increased expression of FURIN and BDNF in the serum of glioma patients after radiotherapy (* *p* value <0.05 vs. GG group; ** *p* value <0.05 vs. GA group). (A) No obvious difference was observed for the expression of miR‐338‐3p in the serum of glioma patients with distinct genotypes at rs4702 after radiotherapy. (B) Rs4702‐A was associated with increased expression of FURIN in the serum of glioma patients after radiotherapy. (C) Rs4702‐A was associated with increased expression of BDNF in the serum of glioma patients after radiotherapy

As shown in Table 1, no obvious difference was observed in respect to the clinical characteristics including sex, right‐handed, Caucasian, age, education, estimated verbal IQ, tumour type and predominant tumour side were compared among the three groups. No significant difference was found in respect to the expression of miR‐338‐3p in the serum of patients with GG, GA and AA genotypes at rs4702 (Figure 1A). In addition, the abundance of FURIN and BDNF was gradually increased in the serum of patients with GA (Figure 1B) and AA (Figure 1C) genotypes when compared with patients with GG genotypes at rs4702. These results indicated that rs4702‐A was associated with enhanced expression of FURIN and BDNF in the serum of glioma patients after radiotherapy treatment.

**TABLE 1:** Basic information of recruited patients

Characteristics	GG (*N* = 38)	GA (*N* = 32)	AA (*N* = 36)	*p* value
Age, years	56.1 ± 5.2	60.0 ± 6.6	57.1 ± 6.8	0.196
Sex, male (%)	26 (68.4)	20 (62.5)	23 (63.9)	0.808
Right handed, n	30 (78.9)	28 (87.5)	29 (80.6)	0.385
Caucaslan, n	28 (73.7)	26 (81.3)	30 (83.3)	0.227
Mean education, y	18.8 ± 6.4	18.6 ± 6.9	17.9 ± 5.3	0.742
Mean estimated verbal IQ	112.8 ± 6.9	109.5 ± 8.3	110.8 ± 10.5	0.820
Tumour type				0.652
Low grade glioma	8 (21.1)	7 (21.9)	8 (22.2)	
High grade glioma	10 (26.3)	10 (31.3)	10 (27.8)	
Primary grade glioma	11 (28.9)	8 (25.0)	10 (27.8)	
Other	9 (23.7)	7 (23.8)	8 (22.2)	
Predominant tumour side				0.458
Left	11 (28.9)	7 (21.9)	8 (22.2)	
Right	18 (47.4)	18 (56.2)	21 (58.3)	
Bilateral	9 (23.7)	7 (21.9)	7 (19.5)	

### 3.2 rs4702‐A was associated with increased expression of FURIN and BDNF in the PBMC of glioma patients after radiotherapy

**FIGURE 2:**
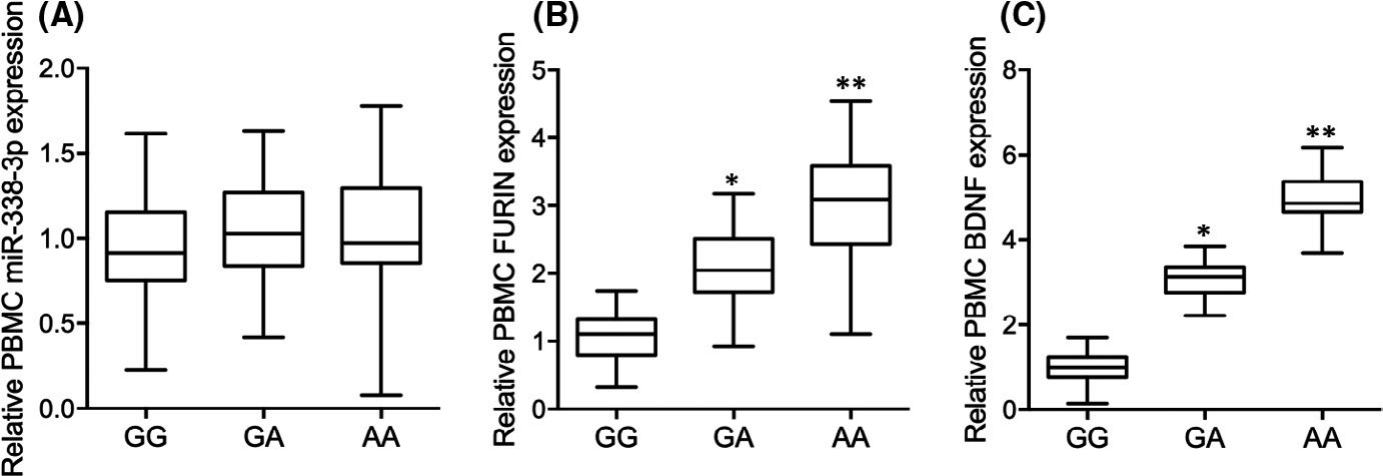
Rs4702‐A was associated with increased expression of FURIN and BDNF in the PBMC of glioma patients after radiotherapy (* *p* value <0.05 vs. GG group; ** *p* value <0.05 vs. GA group). (A) No obvious difference was observed for the expression of miR‐338‐3p in the PBMC of glioma patients with distinct genotypes at rs4702 after radiotherapy. (B) Rs4702‐A was associated with increased expression of FURIN in the PBMC of glioma patients after radiotherapy. (C) Rs4702‐A was associated with increased expression of BDNF in the PBMC of glioma patients after radiotherapy

No significant difference was found for the expression of miR‐338‐3p in the PBMC of patients with GG, GA and AA genotypes at rs4702 (Figure 2A). The abundance of FURIN and BDNF was gradually increased in the PBMC of patients with GA (Figure 2B) and AA (Figure 2C) genotypes when compared with patients with GG genotypes at rs4702. These results indicated that rs4702‐A was associated with enhanced expression of FURIN and BDNF in the PBMC of glioma patients after radiotherapy treatment.

### 3.3 rs4702‐A was associated with increased enterotype I and decreased enterotype III in the stool of glioma patients after radiotherapy

**FIGURE 3:**
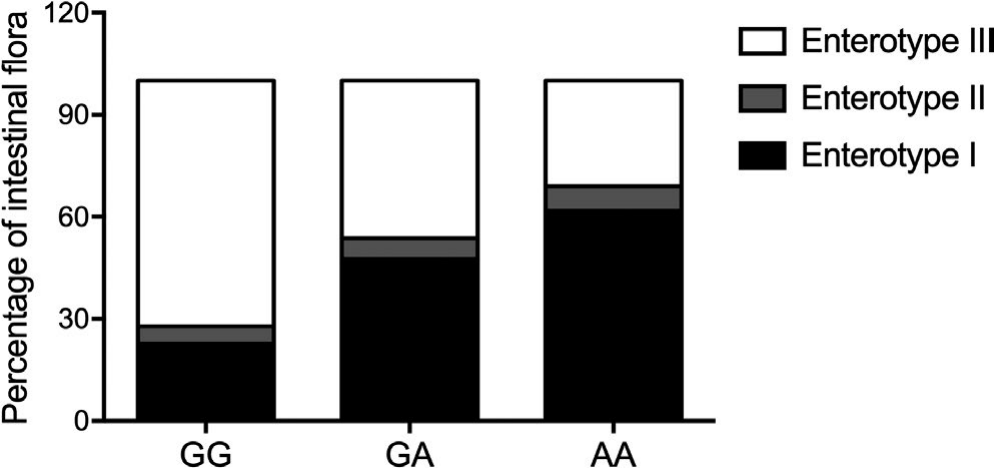
Rs4702‐A was associated with increased enterotype I and decreased enterotype III in the stool of glioma patients after radiotherapy

The percentage of enterotype I was significantly increased for patients carrying A allele (GG: 22.6%, GA: 47.5% and AA: 61.8%), while the percentage of enterotype III was remarkably decreased for patients carrying A allele (GG: 72.2%, GA: 46.2% and AA: 30.9%). In addition, the percentage of enterotype II remained stable in each patient group (GG: 5.2%; GA: 6.3%; and AA: 7.3%). Therefore, the number of A allele was apparently associated with the difference in intestinal flora percentage (Figure 3).

### 3.4 Rs4702‐A was associated with increased MMSE, role functioning and social functioning at three months after radiotherapy

**FIGURE 4:**
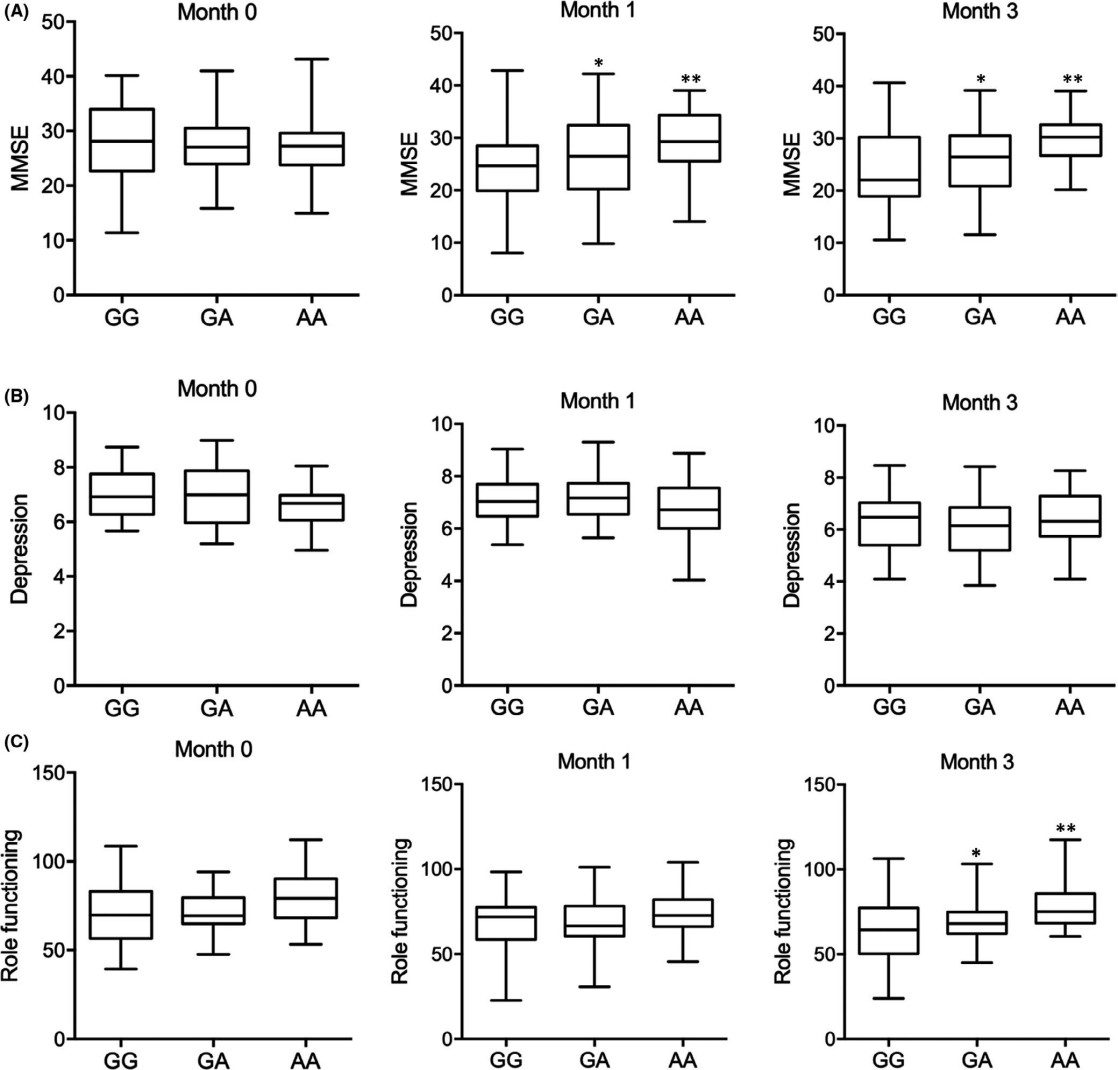
Rs4702 polymorphism was associated with the MMSE, depression and role functioning of glioma patients after radiotherapy (* *p* value <0.05 vs. GG group; ** *p* value <0.05 vs. GA group). (A) Rs4702‐A was associated with increased MMSE of glioma patients at one and three months after radiotherapy. (B) No obvious correlation was observed for the rs4702 polymorphism and depression for glioma patients after radiotherapy. (C) Rs4702‐A was associated with increased role functioning of glioma patients at three months after radiotherapy

**FIGURE 5:**
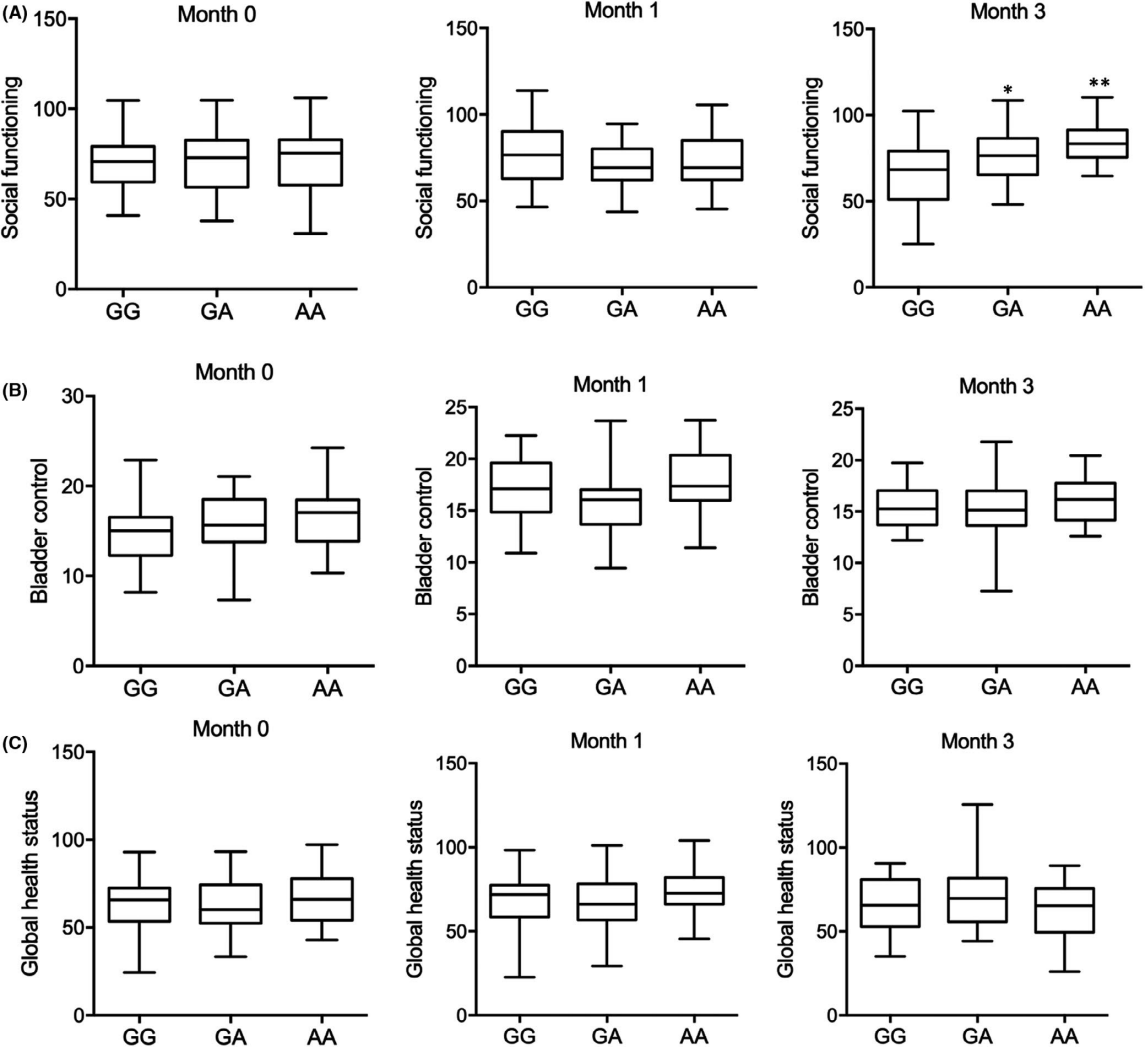
Rs4702 polymorphism was associated with the social functioning, Bladder control and global health status of glioma patients after radiotherapy (* *p* value <0.05 vs. GG group; ** *p* value <0.05 vs. GA group). (A) Rs4702‐A was associated with increased social functioning of glioma patients at 3 months after radiotherapy. (B) No obvious correlation was observed for the rs4702 polymorphism and Bladder control for glioma patients after radiotherapy. (C) No obvious correlation was observed for the rs4702 polymorphism and global health status for glioma patients after radiotherapy

No obvious difference was found for MMSE in patients with distinct genotypes before radiotherapy. The scores for MMSE were remarkably increased at one month and three months after radiotherapy in patients carrying A allele when compared with patients carrying G allele (Figure 4A). No difference was found in depression evaluation for patients with distinct genotypes at rs4702 either before or after radiotherapy (Figure 4B). However, although no difference was found in role functioning assessment (Figure 4C) and social functioning (Figure 5A) at 1 month after radiotherapy, the score of role functioning (Figure 4C) and social functioning (Figure 5A) was obviously increased in patients carrying A allele at 3 months after radiotherapy. Also, no remarkable difference was observed in respect to bladder control (Figure 5B) and global health status (Figure 5C) for patients with distinct genotypes at rs4702 either before or after radiotherapy.

### 3.5 MiR‐338‐3p repressed the luciferase activity of FURIN‐G in U251 and SYSH‐S5 cells

**FIGURE 6:**
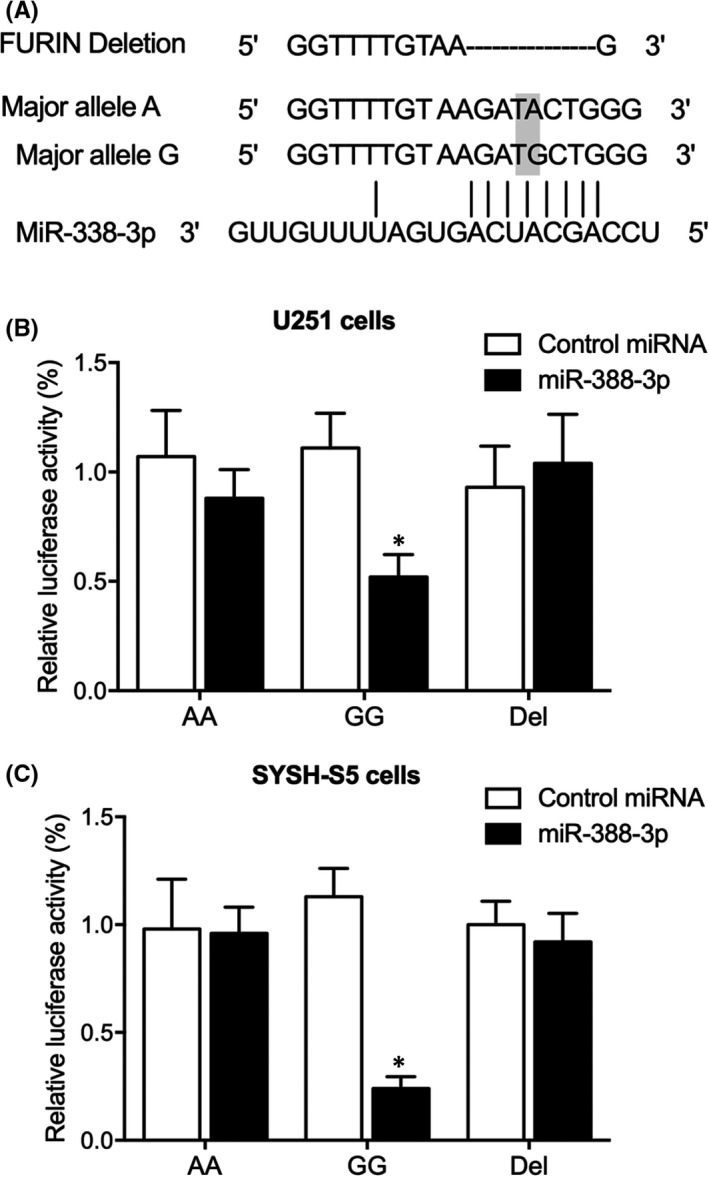
MiR‐338‐3p repressed the luciferase activity of FURIN‐G in U251 and SYSH‐S5 cells (* *p* value <0.05 vs. control miRNA). (A) Sequence analysis by TargetScan (http://www.targetscan.org/) indicated potential binding of miR‐338‐3p to FURIN. (B) Luciferase activities of FURIN‐G were notably suppressed by miR‐338‐3p in U251 cells. (C) Luciferase activities of FURIN‐G were notably suppressed by miR‐338‐3p in SYSH‐S5 cells

As shown in Figure 6A, miR‐338‐3p could bind to the 3’ UTR of FURIN. In addition, the luciferase activities of FURIN‐G vector were remarkably suppressed by miR‐338‐3p while the luciferase activities of FURIN‐A and FURIN‐del vectors were not affected in U251 (Figure 6B) and SYSH‐S5 cells (Figure 6C).

## 4 DISCUSSION

In this study, glioma patients who received radiotherapy were recruited and divided them into three groups based on the rs4702 polymorphisms. Rs4702‐A was significantly associated with increased expression of FURIN and BDNF in the serum and PBMC of glioma patients after radiotherapy.

In addition, the intestinal flora in the stool of glioma patients with distinct genotypes at rs4702 after radiotherapy was evaluated. Rs4702‐A was associated with increased enterotype I and decreased enterotype III in the stool of glioma patients after radiotherapy.

A connection between depression and gut microbiome in human has attracted attention of several researchers. It was suggested that gut microbiome can regulate various brain functions and pathophysiology, such as dementia. Even though its role in dementia has been proposed, a clear mechanism of action is still unknown. In patients with Alzheimer's disease, bacterial lipopolysaccharide was found within the brain lysate, especially in hippocampus and superior temporal lobe.

Mostly, the gut microbiome performs beneficial roles in the body; however, certain unfavourable conditions put stress on the microbiome and can become harmful. Recently, plethora of studies have shown that dysfunction of microorganism in the intestines could be responsible for age related lowering of brain functions, such as memory and learning. Moreover, a study found that administrating probiotics and prebiotics for three months resulted in improvement in brain functions of Alzheimer's disease patients.^7^

Several neuronal functions are regulated by miR‐338‐5p including plasticity and outgrowth.^8^ In addition, miR‐338‐5p when stimulated may lead to improvement to spinal cord after an injury, and on the contrary, lowered expression of miR‐338‐5p could contribute to the development of AD. In this study, the protective effect of miR‐338‐5p on decreased cognitive functions using mice with genetically modified APP/PS1 gene was investigated. Here, the authors have demonstrated that the microRNA could negatively modulate the advancement of AD and offer an innovative strategy to treat the debilitating disease.^8^, ^15^

Recently, it was proven that the proprotein convertase, named FURIN, was very efficient at converting pro‐BDNF to BDNF. Like FURIN, PACE4 and PC5/6‐B can successfully cleave pro‐BDNF to some extent.^16^ The results of the manuscript provide evidence for maturation of BDNF involves FURIN cleavage. Inhibition of FURIN by ppFURIN reduces concentration of BDNF both within the cellular compartments and top clear layer of astrocytes culture.

Several physiological processes inside brain, such as plasticity of synapses, cognition and generation of new nerve cells, are governed by brain‐derived neurotrophic factor (BDNF), which is known to be upregulated during severe exercise.^17^ Downstream signalling pathways activated by BDNF lead to eventual activation of transcription factor cAMP‐calcium response element binding protein (CREB). Phosphorylation of CREB is involved in activation of transcription of several genes, which are key for neuron survival and functions.^18^

Previous studies, both in animals and humans, have provided insight into the relationship between gut microbiome and expression of several receptors or transporters in brain. This important relationship of gut‐brain axis dictates expression of 5‐hydroxytryptophan (5‐HT) transporter and neurotropin (NT) growth factor along with the brain‐derived neurotrophic factor (BDNF).^19^, ^20^, ^21^ Additionally, clinical studies have reported that changes in gut microbiota lead to observable changes in mood or behaviour, while conversely, ingestion of probiotics can positively affect brain function in healthy individuals.^22^, ^23^, ^24^ We have found that following radiotherapy, rs4702‐A is significantly correlated to social and role functioning.

In hippocampus, downregulation of BDNF is driven by binding of 3‐UTR by microRNA miR‐34a‐5p. miR‐34a‐5p is upregulated in small intestine and peripheral blood by administration of the microRNA by tail intravenous injection. Subsequently, intestinal microbiome is preserved by the miR‐34a‐5p antagomir injection.^10^ The results of this study provided evidence that the antibiotic group showed increased number of Class‐Gammaproteobacteria, which are proinflammatory compared to the control group. In addition, the authors also carried out the luciferase assay to investigate the role of miR‐338‐3p on FURIN expression levels.

Due to almost impermeable barrier called blood‐brain barrier, large and hydrophilic molecules are unable to enter CNS,^25^ while peripheral changes of miRNAs were found to be associated with psychiatric illness, which suggested that circulating miRNAs affect the function of brain.^26^ Therefore, the authors have utilized miRNAs to investigate mechanism of the reduction in cognition by TAI.

### 4.1 Limitation

The sample size recruited is limited in our study. In addition, animal experiment should be performed to further validate our findings.

## 5 CONCLUSION

Compared with G allele, the presence of A allele of rs4702 polymorphism in FURIN mRNA could obstruct the suppressive effect of miR‐338‐3p upon FURIN and BDNF by disrupting their binding. Therefore, the carriers of A allele, that is glioma patients with GA or AA genotype of rs4702, are at less risk of radiotherapy‐induced cognitive impairment.

## CONFLICT OF INTEREST

None.

## AUTHOR CONTRIBUTIONS

**Sen Yang:** Conceptualization (equal); Formal analysis (equal); Resources (equal); Writing – original draft (equal). **Zhanzhao Fu:** Conceptualization (equal); Data curation (equal). **Yanqiu Zhang:** Data curation (equal); Resources (equal). **Baohong Fu:** Investigation (equal). **Lixin Dong:** Conceptualization (equal); Data curation (equal); Formal analysis (equal); Resources (equal); Software (equal); Supervision (equal); Validation (equal); Visualization (equal); Writing – original draft (equal).

## Data Availability

The data that support the findings of this study are available from the corresponding author upon reasonable request.
